# Neutron capture measurements for s-process nucleosynthesis: A review about CERN n_TOF developments and contributions

**DOI:** 10.1140/epja/s10050-025-01563-z

**Published:** 2025-05-19

**Authors:** The n_TOF Collaboration, C. Domingo-Pardo, O. Aberle, V. Alcayne, G. Alpar, M. Al Halabi, S. Amaducci, V. Babiano, M. Bacak, J. Balibrea-Correa, J. Bartolomé, A. P. Bernardes, B. Bernardino Gameiro, E. Berthoumieux, R. Beyer, M. Birch, M. Boromiza, D. Bosnar, B. Brusasco, M. Caamaño, A. Cahuzac, F. Calviño, M. Calviani, D. Cano-Ott, A. Casanovas, D. M. Castelluccio, D. Catlett, F. Cerutti, G. Cescutti, E. Chiaveri, G. Claps, P. Colombetti, N. Colonna, P. Console Camprini, G. Cortés, M. A. Cortés-Giraldo, L. Cosentino, S. Cristallo, A. D’Ottavi, G. de la Fuente Rosales, S. F. Dellmann, M. Diakaki, M. Di Castro, A. Di Chicco, M. Dietz, E. Dupont, I. Durán, Z. Eleme, M. Eslami, S. Fargier, B. Fernández-Domínguez, P. Finocchiaro, W. Flanagan, V. Furman, A. Gandhi, F. García-Infantes, A. Gawlik-Ramiega, G. Gervino, S. Gilardoni, E. González-Romero, S. Goula, E. Griesmayer, C. Guerrero, F. Gunsing, C. Gustavino, J. Heyse, W. Hillman, D. G. Jenkins, E. Jericha, A. Junghans, Y. Kadi, K. Kaperoni, I. Kelly, M. Kokkoris, Y. Kopatch, M. Krtička, N. Kyritsis, C. Lederer-Woods, J. Lerendegui-Marco, A. Manna, T. Martínez, M. Martínez-Cañada, A. Masi, C. Massimi, P. Mastinu, M. Mastromarco, E. A. Maugeri, A. Mazzone, E. Mendoza, A. Mengoni, V. Michalopoulou, P. M. Milazzo, J. Moldenhauer, R. Mucciola, E. Musacchio González, A. Musumarra, A. Negret, E. Odusina, D. Papanikolaou, N. Patronis, J. A. Pavón-Rodríguez, M. G. Pellegriti, P. Pérez-Maroto, A. Pérez de Rada Fiol, G. Perfetto, J. Perkowski, C. Petrone, N. Pieretti, L. Piersanti, E. Pirovano, I. Porras, J. Praena, J. M. Quesada, R. Reifarth, D. Rochman, Y. Romanets, A. Rooney, G. Rovira, C. Rubbia, A. Sánchez-Caballero, R. N. Sahoo, D. Scarpa, P. Schillebeeckx, A. G. Smith, N. V. Sosnin, M. Spelta, M. E. Stamati, K. Stasiak, G. Tagliente, A. Tarifeño-Saldivia, D. Tarrío, P. Torres-Sánchez, S. Tosi, G. Tsiledakis, S. Valenta, P. Vaz, G. Vecchio, D. Vescovi, V. Vlachoudis, R. Vlastou, A. Wallner, C. Weiss, P. J. Woods, T. Wright, R. Wu, P. Žugec

**Affiliations:** 1 Instituto de Física Corpuscular, CSIC, Universidad de Valencia, Valencia, Spain; 2 European Organization for Nuclear Research (CERN), Geneva, Switzerland; 3 Centro de Investigaciones Energéticas Medioambientales y Tecnológicas (CIEMAT), Madrid, Spain; 4 University of Dallas, Irving, USA; 5 Goethe University Frankfurt, Frankfurt, Germany; 6 INFN Laboratori Nazionali del Sud, Catania, Italy; 7 Universitat Politècnica de Catalunya, Barcelona, Spain; 8 University of Manchester, Manchester, UK; 9 Universidad de Sevilla, Seville, Spain; 10 CEA Irfu, Université Paris-Saclay, 91191 Gif-sur-Yvette, France; 11 Helmholtz-Zentrum Dresden-Rossendorf, Dresden, Germany; 12 Horia Hulubei National Institute of Physics and Nuclear Engineering, Măgurele, Romania; 13 Department of Physics, Faculty of Science, University of Zagreb, Zagreb, Croatia; 14 University of Santiago de Compostela, Santiago, Spain; 15 Agenzia nazionale per le nuove tecnologie, l’energia e lo sviluppo economico sostenibile (ENEA), Brindisi, Italy; 16 Istituto Nazionale di Fisica Nucleare, Sezione di Bologna, Bologna, Italy; 17 Istituto Nazionale di Fisica Nucleare, Sezione di Trieste, Trieste, Italy; 18 Department of Physics, University of Trieste, Trieste, Italy; 19 INFN Laboratori Nazionali di Frascati, Frascati, Italy; 20 Istituto Nazionale di Fisica Nucleare, Sezione di Torino, Turin, Italy; 21 Department of Physics, University of Torino, Turin, Italy; 22 Istituto Nazionale di Fisica Nucleare, Sezione di Bari, Bari, Italy; 23 Istituto Nazionale di Fisica Nucleare, Sezione di Perugia, Perugia, Italy; 24 Istituto Nazionale di Astrofisica, Osservatorio Astronomico d’Abruzzo, Abruzzo, Italy; 25 National Technical University of Athens, Athens, Greece; 26 Physikalisch-Technische Bundesanstalt (PTB), Bundesallee 100, 38116 Braunschweig, Germany; 27 University of Ioannina, Ioannina, Greece; 28 University of York, York, UK; 29 Affiliated with an Institute Covered by a Cooperation Agreement with CERN, Meyrin, Switzerland; 30 University of Granada, Granada, Spain; 31 University of Lodz, Lodz, Poland; 32 TU Wien, Atominstitut, Stadionallee 2, 1020 Wien, Austria; 33 Istituto Nazionale di Fisica Nucleare, Sezione di Roma 1, Rome, Italy; 34 European Commission, Joint Research Centre (JRC), Geel, Belgium; 35 Charles University, Prague, Czech Republic; 36 School of Physics and Astronomy, University of Edinburgh, Edinburgh, UK; 37 Dipartimento di Fisica e Astronomia, Università di Bologna, Bologna, Italy; 38 INFN Laboratori Nazionali di Legnaro, Legnaro, Italy; 39 Dipartimento Interateneo di Fisica, Università degli Studi di Bari, Bari, Italy; 40 Paul Scherrer Institut (PSI), Villigen, Switzerland; 41 Consiglio Nazionale delle Ricerche, Bari, Italy; 42 Istituto Nazionale di Fisica Nucleare, Sezione di Catania, Catania, Italy; 43 Department of Physics and Astronomy, University of Catania, Catania, Italy; 44 Instituto Superior Técnico, Lisbon, Portugal; 45 Japan Atomic Energy Agency (JAEA), Tokai-Mura, Naka, Japan; 46 Department of Physics and Astronomy, Uppsala University, Box 516, 75120 Uppsala, Sweden

## Abstract

This article presents a review about the main CERN n_TOF contributions to the field of neutron-capture experiments of interest for *s*-process nucleosynthesis studies over the last 25 years, with a special focus on the measurement of radioactive isotopes. A few recent capture experiments on stable isotopes of astrophysical interest are also discussed. Results on *s*-process branching nuclei are appropriate to illustrate how advances in detection systems and upgrades in the facility have enabled increasingly challenging experiments and, as a consequence, have led to a better understanding and modeling of the *s*-process mechanism of nucleosynthesis. New endeavors combining radioactive-ion beams from ISOLDE for the production of radioisotopically pure samples for activation experiments at the new NEAR facility at n_TOF are briefly discussed. On the basis of these new exciting results, also current limitations of state-of-the-art TOF and activation techniques will be depicted, thereby showing the pressing need for further upgrades and enhancements on both facilities and detection systems. A brief account of the potential technique based on inverse kinematics for direct neutron-capture measurements is also presented.

## Introduction

Neutron-capture reactions play a fundamental role in the cosmic origin of elements heavier than iron, both during hydrostatic stages of stellar evolution (*s*-process) and in cataclysmic stellar environments (*r*-process). These two nucleosynthesis mechanisms were first presented in detail in the works of B2FH [1] and Cameron [2]. As a consequence, a large experimental effort over the last 70 years has resulted in a wealth of neutron-capture nuclear data. The first experiments were initially aimed at validating the *s*-process hypothesis [3, 4], and shortly afterwards to aid in the development and refinement of stellar models while constraining the physical conditions along different evolutionary stages of stars [5, 6]. One can see also the review of Käppeler et al. [7] and references therein. Two different methodologies for neutron-capture cross-section measurements have been extensively applied so far in many laboratories worldwide, neutron time-of-flight (TOF) and neutron activation, thereby covering about 350 (predominantly stable) nuclei [8]. However, there are still acute needs for new neutron-capture cross-section measurements, and for a large fraction of the measured isotopes improvements are required both in terms of accuracy (± 5%) and energy-range completeness (1 eV up to 100 keV).

There are three “families” of nuclides with a particular value for *s*-process studies. The group of *s*-only nuclei serves as a benchmark for *s*-process and galactic-chemical evolution (GCE) models, which should reproduce 100% of the *s*-only isotopic abundances [9–11]. The group of *s*-process bottlenecks gives rise to the three characteristic *s*-process abundance peaks at the neutron-shell closures N = 50, 82 and 126. Because of their large abundances, elements in the bottleneck peaks show-up prominently in spectroscopic observations of stellar atmospheres and thus, they represent a sensible probe for stellar models. Finally, *s*-process branching nuclei are especially relevant for constraining the physical conditions of the stellar environment [7]. Interestingly, for none of these groups the quality of the data complies with the ± 5% uncertainty level required by stellar models. The neutron-capture cross sections of most *s*-only nuclei are relatively well determined [8], which does not necessarily imply that their *s*-process abundances are correspondingly accurate. This is a consequence of the strong interplay between many *s*-process branchings and *s*-only isotopes [10, 12]. The astrophysical impact of the uncertainties on the *s*-process branchings is therefore twofold, because the final *s*-only abundances are important both for benchmarking the performance of stellar models [13] and for deriving the isotopic *r*-process abundance distributions in the solar system [11, 14, 15].

Past and ongoing efforts at CERN n_TOF to improve this situation are discussed in this article. A large number of articles already describe the n_TOF facility and the related measurement techniques in great detail [16–20] and thus, only some main facility features relevant for the discussions in the present article will be summarized in Sect. 2. Some of the main challenges in neutron-capture TOF experiments are related to the quality (isotopic enrichment) and quantity of sample material available for the experiment. In some cases, the highly-sensitive neutron-activation technique may be the only option when the number of sample atoms is very limited or the enrichment very low for a TOF measurement. For isotopes with very short half-lives surrogate reactions [21] or other indirect techniques, like the $\beta$-Oslo method [22] may provide unique information on the capture channel of interest. However, most of the unstable isotopes of interest for *s*-process nucleosynthesis correspond to branching points with relatively long terrestrial half-lives in the range of $\sim$ 1–100 years, or longer. Because of their relevance for *s*-process nucleosynthesis and the demanding aspects of these experiments, measurements on unstable *s*-process branching nuclei are well suited to illustrate the experimental progress achieved over the last 25 years. Thus, Sect. 3 presents in chronological order the main results on *s*-process branching nuclei measured at n_TOF, along with the facility and detector advances that were relevant for such studies. Measurements on stable isotopes involved in the *s*-process path are also of paramount importance for properly interpreting the observed elemental abundances in stars and the isotopic composition of meteorites, and thus for a better understanding of the *s*-process mechanism. Some recent examples will be discussed in Sect. 4. Finally, Sect. 5 summarizes some of the main limitations of current state-of-the-art TOF experiments, and presents ongoing efforts to improve present instruments and to complement them with new measuring stations and techniques.

## The n_TOF facility

**Fig. 1:**
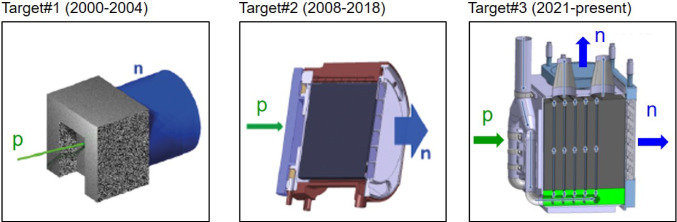
From left to right, the three generations of spallation sources at n_TOF. The latest version [24] delivers a superior neutron-beam quality for capture experiments at both measuring stations EAR1 (185 m) and EAR2 (20 m) [25]

n_TOF utilizes a 6 ns wide, 20 GeV pulsed proton beam from CERN’s Proton Synchrotron (PS), typically delivering 8.5$\times$10$^{12}$ protons per pulse to a lead spallation target (Fig. 1), where approximately 300 neutrons are produced per incident proton. A beam line with a length of $\sim$185 m leads to an experimental area (EAR1), where samples and detectors are mounted for neutron-induced reaction experiments. More details about EAR1 can be found in Ref. [18] and references therein. The neutron energy is determined with a high precision by means of the time-of-flight technique [23]. The low duty cycle and the high instantaneous flux are two of the most remarkable facility features. Neutron bunches are spaced at intervals of at least 1.2 s, corresponding to the operational cycle of the CERN PS. This duty cycle enables measurements over an extended TOF span, facilitating the detection of low-energy neutrons without overlap from subsequent neutron cycles. As a result, neutron energies as low as approximately 10 meV can be measured, with the high-energy portion of the spectrum remaining unaffected by slow neutrons from previous cycles. The exceptionally high instantaneous neutron flux is particularly advantageous when working with radioactive samples, as it ensures a highly favorable ratio of neutron-induced reaction signals to background signals caused by radioactive decay events.

At the core of the facility, the optimization of the spallation target has been one of the key aspects for the successful realization of neutron-capture experiments (see Fig.  1). A layer of water moderates the initially fast neutrons into a white spectrum covering a broad energy range from meV to GeV, thus fully including the energy-range of astrophysical interest for *s*-process studies ($\sim$1 eV–100 keV). During phase-I (2001–2004) a water layer served as coolant and moderator for an 80$\times$80$\times$60 cm$^3$ lead target (Fig. 1 left). This design severely limited the lifetime of the target owing to corrosion effects and, more relevant for capture experiments, contaminant neutron-capture reactions in the hydrogen of the water induced a large in-beam $\gamma$-ray (E$_\gamma$ = 2.2 MeV) background. The latter was a minor issue for the measurement of isotopes with large capture cross sections, like unstable $^{151}$Sm [26], but it made quite difficult the measurement of neutron-magic isotopes with small capture cross sections, like $^{208}$Pb or $^{209}$Bi [27]. A new cylindrical (40 cm length, 60 cm diameter) target design (see Fig. 1 middle) incorporated several improvements for phase-II (2009–2012), among them the use of an 1 cm water cooling circuit and a separate $^{10}$B-loaded 4-cm thick demineralized-water moderator, which efficiently suppressed the in-beam 2.2 MeV $\gamma$-ray background. However, during the last years of operation an increase in the activity of the cooling circuit was detected, which was ascribed to corrosion in the lead-water interface. In 2014 a second experimental area EAR2 was constructed at only 20 m above the already existing spallation target [28, 29]. The new measuring station allowed one to significantly extend the number of experiments during phase-III (2014–2018) (see Fig. 1 in Ref. [20]). Initially, the large instantaneous flux of EAR2 could not be efficiently utilized for *s*-process capture studies because the target geometry and moderator characteristics were not optimal for the new EAR2 station [30, 31]. However, all these limitations were remarkably improved in phase-IV (2021–) with the third-generation neutron target [24] (see Fig. 1 right). With the new target a slightly higher neutron flux was achieved and, more importantly, the resolution function (RF) at EAR2 could be significantly enhanced (see Fig.2 in [25]). The number of neutrons per proton pulse reaching EAR1 and EAR2 are 10$^6$ n/cm$^2$ and 10$^{8}$ n/cm$^2$, respectively, while the relative neutron-energy resolution at E$_n \sim 1$ keV is of 0.05% (0.8%) in EAR1 (EAR2). More details about the n_TOF neutron beams and a comparison with respect to other facilities can be found in Ref. [19]. The large instantaneous neutron flux and narrow resolution function at n_TOF have become of special importance for astrophysics experiments [32] and, in particular, the new target features enabled at EAR2 some of the most challenging and fascinating neutron-capture TOF experiments performed so far, as it is discussed in Sect. 3.

Two different detection systems and techniques are regularly used at CERN n_TOF for determining neutron-capture cross sections. The first is a Total Absorption Calorimeter (TAC), composed of 40 BaF$_2$ crystals [33], installed in EAR1. This system enables neutron-capture cross-section measurements using the total $\gamma$-ray absorption method [34]. The second method, based on the total energy detection (TED) principle [35], is applied both in EAR1 and EAR2. In this case radiative neutron-capture reactions are measured using low-efficiency scintillation detectors combined with the Pulse-Height Weighting Technique (PHWT) [5]. All the measurements presented in this article rely on the TED principle and the PHWT. A Monte-Carlo based analysis methodology enables remarkable systematic accuracy with the PHWT ($2\%$ rms) [36, 37]. One key advantage of the PHWT is its flexibility in detector design, allowing continuous improvements in detection sensitivity, as it is described in Sects. 3 and 4.

## Progress on *s*-process branching isotopes

Unstable nuclei with relatively long half-lives ($\sim$years) are particularly interesting for *s*-process studies because they create a branching in the *s*-process path. The strength of the branching determines the local isotopic pattern around the unstable nucleus, making the resulting isotopic abundances sensitive to the physical conditions in thermally-pulsing asymptotic giant-branch (TP-AGB) stars with masses between 1 and 3 solar masses (1M$_{\odot } \le$ M $\le 3$M$_{\odot }$) and in massive stars with M $\ge 8$M$_{\odot }$ [7]. Therefore, accurate neutron-capture cross-section measurements of these nuclides, combined with isotopic analysis of meteorites and advanced stellar models, can provide valuable constraints on the temperature and neutron-density conditions that govern the *s*-process mechanism of nucleosynthesis. From the experimental viewpoint, producing (radioactive) samples with a sufficient number of atoms ($\ge 10^{18}$ atoms), and with a high enough enrichment becomes a true challenge. For most of the experiments discussed in this section a sample-production strategy based on the combined effort from Institut Laue-Langevin (ILL)-Grenoble (France) and Paul Scherrer Institute (PSI)-Villigen (Switzerland) was pursued. At ILL isotope-production via thermal-neutron activation was carried out, while the radiochemistry laboratory of PSI produced the samples for irradiation at ILL and in some cases performed a posterior radiochemical separation and purification. Still, many of the measured radioactive samples posed important challenges for the capture experiment, which were mainly related to the sample radioactivity, to the final number of atoms available for the isotope of interest and to the sample purity or enrichment.

In order to cope with such demanding cases, a big effort has been made over the last 25 years at CERN n_TOF for optimizing the quality of the neutron beam in terms of resolution function, high instantaneous neutron flux and low gamma- and neutron-induced backgrounds. In addition, both continuous adaptations and disruptive approaches have been pursued with the detection systems [38–45], leading to a progressive increase in detection sensitivity for the radiative neutron-capture channel of astrophysical interest. These advances are described in more detail below.

**Fig. 2:**
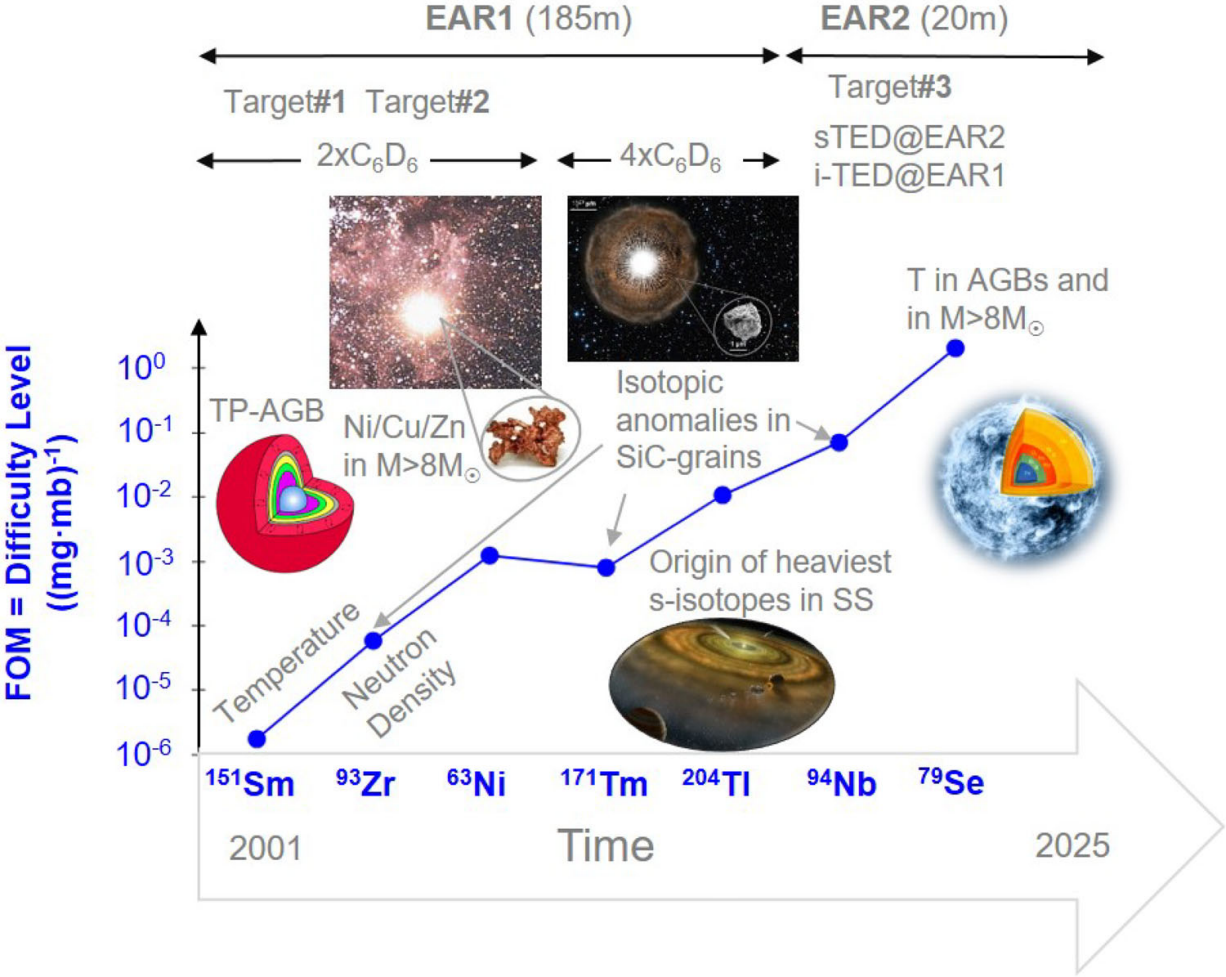
Over the past two decades, advancements in facility upgrades (EAR1, EAR2, Target#1-3) and detection systems (C$_6$D$_6$, sTED, i-TED) at CERN n_TOF have enabled increasingly challenging experiments on *s*-process branching nuclei (horizontal axis). The figure of merit (FOM, vertical axis), defined in Eq. (1), provides a visual representation of this progress. For details on various *s*-process branching cross-section measurements, refer to the main text. Additional information on Target#1–3 can be found in Fig. 1, while details on detection systems are in Figs. 3 and 4. Abbreviations: temperature (T), Solar System (SS), Thermally-Pulsing Asymptotic Giant-Branch star (TP-AGB). Credits for figure insets: TP-AGB drawing adapted from [46]; Hubble-Space Telescope image of a TP-AGB (U. Camelopardalis) with a SiC grain (NASA/Nan Liu/Andrew Davis), massive-star illustration (NASA/CXC/M. Weiss)

Such progress can be well illustrated via the measured *s*-process branching isotopes and the corresponding facility and detector upgrades. To do so, a figure of merit (FOM) is arbitrarily defined here as

1 $$\begin{aligned} FOM = \frac{1}{m_i \cdot \sigma _i \cdot f_e}, \end{aligned}$$

where $m_i$ is the mass of the isotope of interest in the sample (in mg), $\sigma _i$ is the Maxwellian averaged capture cross section (MACS) at 30 keV (in mb), and $f_e$ is the enrichment factor for the isotope of interest in the sample. This FOM will be also referred to as “difficulty level” along this article, and it is shown in Fig. 2 as a function of the *s*-process branching isotope measured along the different years. At this time, final results have been published for all these cases except for $^{94}$Nb($n,\gamma$) and $^{79}$Se($n,\gamma$), which are still undergoing data analysis. In short, the smaller are the sample mass, the cross section and the enrichment, the more difficult it becomes to perform the measurement. The FOM does not include the half-life of the isotope, nor the difficulty ascribed to the background induced by the sample-decay itself, which can be very different depending on whether there is emission of high-energy $\gamma$-rays or not. However, as we will see, for the cases and the range of terrestrial half-lives discussed here (2–4 Myr) there is no correlation between difficulty and half-life, because the other ingredients became a much more significant constraint for the measurement. Remarkably, as it will be seen later, at least for the cases described here the main limitations in terms of sample-radioactivity were not due to the decay of the isotope of interest itself, but rather to different levels of $^{60}$Co contamination in the samples (see discussion below for $^{204}$Tl, $^{94}$Nb and $^{79}$Se).

A more relevant ingredient, which is not included in the FOM, is the effective neutron-energy range actually covered in the measurement, i.e. the neutron-energy range exploitable in the analysis of the capture data. The radiative neutron-capture cross section decreases with neutron energy as $\sim 1/\sqrt{E_n}$, while $\gamma$-ray backgrounds remain constant or even increase with increasing neutron energy. This aspect is difficult to include in such a FOM and, as we will see, it is worth recognizing that an effort should be made to extend the energy range for most of the measured *s*-process branching points discussed here, once alternative approaches or improved measuring conditions become available in the future.

### $^{151}$Sm($n,\gamma$): temperature in He-shell flashes of low-mass red giant stars

**Fig. 3:**
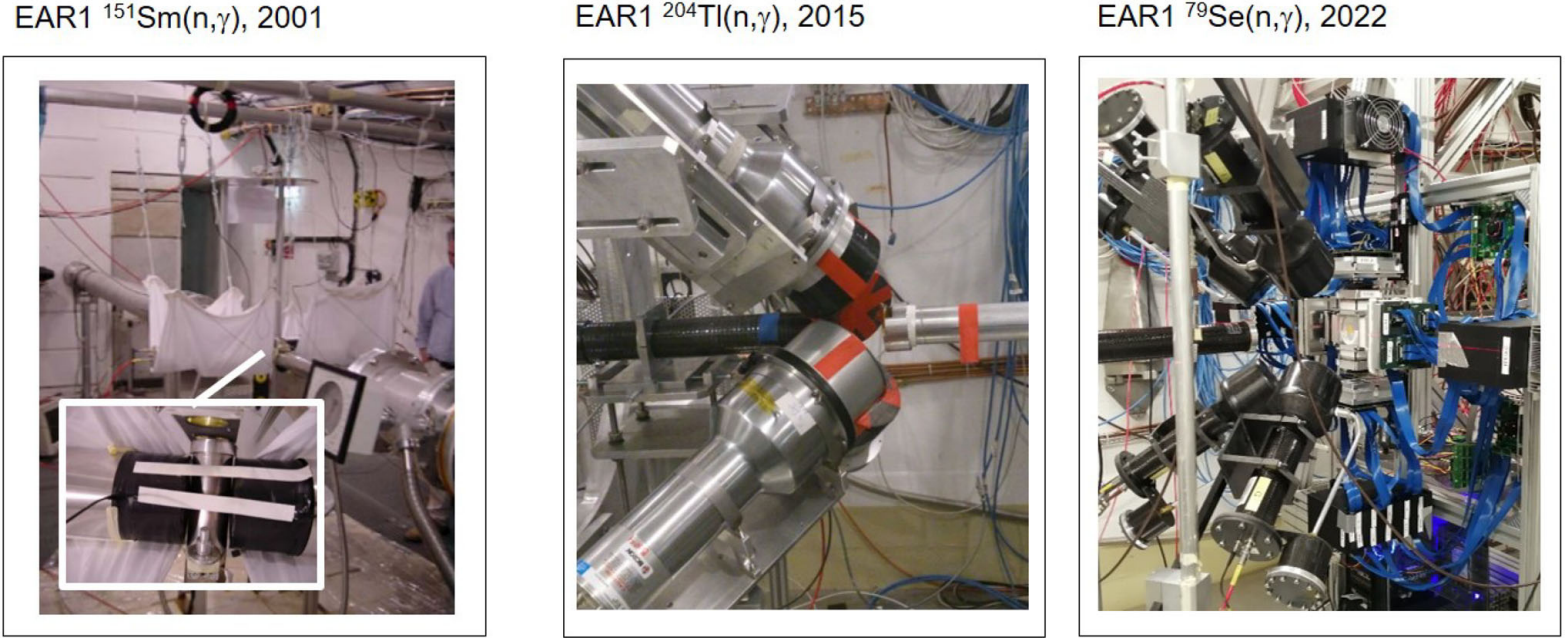
Evolution of the capture set-up at EAR1, from left to right, two first-generation C-fiber based C$_6$D$_6$ detectors for the measurement of $^{151}$Sm($n,\gamma$) [26], four Bicron C$_6$D$_6$ detectors with lead shields for the $^{204}$Tl($n,\gamma$) experiment [63] and four latest-generation C-fiber C$_6$D$_6$ detectors [39] plus the i-TED Compton array [40] for the $^{79}$Se($n,\gamma$) cross-section measurement [43]

$^{151}$Sm (t$_{1/2}$ = 94.6 y) was the very first *s*-process branching measured at the beginning of the n_TOF experiment in 2001 and published in 2004 [26]. A highly enriched ($\sim$90%) sample of $^{151}$Sm with a mass of 206 mg (8$\times$10$^{20}$ atoms) was measured at the EAR1 station using a set of two C$_6$D$_6$ detectors placed at 90$^{\circ }$ with respect to the beam axis at the sample position (see Fig. 3). These detectors were designed with the aim of enhancing detection efficiency and reducing neutron-induced backgrounds [38] (see also discussion in Sect. 4). Both in-beam $\gamma$-rays background and the high sample activity (156 GBq) played a minor role over the entire energy range of interest for this measurement (see Fig. 2 in [26]) due to the large capture cross section ($\sim$3 b at 30 keV) and the fact that $^{151}$Sm decays via pure $\beta$ emission without high-energy $\gamma$-rays. The neutron-capture cross section was determined in the neutron-energy range from 0.6 eV up to 1 MeV. A MACS of 3100(160) mb was obtained at $kT=30$ keV (5% relative uncertainty) [47]. Despite being the very first TOF measurement of the neutron-capture cross section of $^{151}$Sm it was rather straight-forward and, quite remarkably, the neutron-capture cross section could be determined over the full stellar energy range of astrophysical relevance (see Fig. 2 in [26]). None of the *s*-process branching nuclei measured later could replicate this feature due to significantly more demanding sample requirements and experimental conditions.

Although the limitations of the phenomenological *s*-process model [48] were already uncovered many years before thanks to a series of dedicated experiments at the Karlsruhe Van de Graaff accelerator [13], the new n_TOF results on $^{151}$Sm($n,\gamma$) served to confirm such limitations and helped to refine the physical conditions of modern models of TP-AGB stars [49]. In the context of the classical *s*-process [48] the large cross-section values measured at n_TOF in combination with the high neutron density obtained from other neighboring branching nuclei [50] would imply an unrealistic temperature regime, i.e. in excess of 4$\times 10^8$ K, for the *s*-process operating during He-burning in TP-AGB stars. More advanced hydrodynamical models for low-mass TP-AGB stars [49] in combination with the measured cross section helped to constrain the thermal conditions of the He-shell flashes within a range of *T* = 2.5–2.8$\times$10$^8$ K, as well as to assess more consistently the *s*- and *p*-process contributions in the important Sm-Eu-Gd region [47].

### $^{93}$Zr($n,\gamma$): interstellar SiC grains, temperature and neutron density in AGB stars

The measurement of $^{93}$Zr(n,$\gamma$) [51] was significantly more difficult despite of its relatively long half-life of 1.6 My (see Fig. 2). Indeed, the capture cross section of this Zr isotope is relatively small ($\sim$96 mb at 30 keV) and the sample enrichment was of only 20%. Similarly to the measurement of the $^{151}$Sm-branching, two C$_6$D$_6$ detectors were used. In this case they were placed 9 cm upstream in order to reduce the background effect of in-beam $\gamma$-ray Compton scattering in the sample, which has a forward distribution for high-energy $\gamma$-rays. The main limitation in this experiment was due to the background contribution beyond the $\sim$keV neutron-energy range arising from neutrons scattered in the sample and subsequently captured in the surrounding materials (see “setup” labeled spectrum in Fig. 1 of Ref. [51]). As a consequence of this, the neutron-capture cross section could be determined only from 0.6 eV up to $\sim$8 keV. The MACS at $kT=30$ keV was determined with a 9% relative uncertainty as 96(9) mb [51].

Including the new $^{93}$Zr($n,\gamma$) cross section measured at n_TOF in state-of-the-art models for thermally pulsing asymptotic-giant branch (TP-AGB) stars [52] allowed one to get a better insight about the origin of interstellar SiC grains in the CM2 (Mighei type) Murchison chondrite. Chondrites are meteorites that have not been modified by melting or differentiation processes in their parent bodies. These chondrites contain SiC grains, individual micro-crystals that were already present in the protosolar nebula and which have survived from destructive processes in the interplanetary disk, in the parent bodies of their host chondrites, and also on their way to the Earth. Utilizing the cross section measured at n_TOF Lugaro and coworkers [53] found that the composition of the SiC grains arises mainly from low-mass (1.5 $M_\odot \le M \le 4 M_\odot$) TP-AGB stars, and ruled out the possibility of significant contributions from $\ge 4 M_\odot$ AGB stars, where the $^{22}$Ne($\alpha ,n$) source is more efficiently activated than in the lower-mass stars. A series of systematic capture-measurements on the stable $^{90,91,92,94,96}$Zr-isotopes at n_TOF [54–58] in combination with new AGB models [59, 60] allowed to explore even more features about the origin of the SiC grains [61]. It was found that C-rich 1.25–4 M$_\odot$ AGB stars with metallicities in the range of Z=0.01-0.03 reproduced well the variations of $^{90,91}$Zr/$^{94}$Zr measured in the grains, as well as their Si-isotopic composition. The stellar metallicity was found to be the main parameter for explaining the correlation found between $^{92}$Zr/$^{94}$Zr versus $^{29}$Si/$^{28}$Si in these grains, rather than other effects such as stellar rotation [61]. In summary, the complete series of measurements on Zr isotopes allowed one to inspect important open questions about low-mass TP-AGBs, mainly related to the effects of the stellar mass, uncertainties in the $^{13}$C-pocket and the interplay between metallicity and rotation [61]. Finally, the neutron-energy dependence of the cross sections measured at n_TOF [51], together with the abundance ratio N(Nb)/N(Zr) observed in extrinsic S-type red-giant stars, allowed one to directly determine an effective value of the *s*-process temperature ($\lesssim$ 2.5$\times$10$^8$K) in evolved low-mass red-giant stars, independently of stellar evolution models [62]. As discussed in the latter work, if new experiments with improved accuracy (or equivalently with a reduced background in the keV neutron-energy region) could be performed, they would help to constrain even more the *s*-process temperature in red-giant TP-AGB stars (see N(Zr)/N(Nb) error bars in Fig. 1 of [62]).

### $^{63}$Ni($n,\gamma$): constraining Ni-Cu-Zn inventory before CCSN explosion

A similar set-up of two C$_6$D$_6$ detectors placed $\sim$9 cm upstream was used for the measurement of $^{63}$Ni($n,\gamma$) (t$_{1/2}=101$ year) [64]. This experiment implied a step further in difficulty (see Fig. 2) mostly due to the low sample enrichment ($\sim$10%) and the reduced number of $^{63}$Ni atoms in the sample. In spite of the excellent TOF (neutron-energy) resolution provided by the 185 m flight-path of EAR1, strong resonance-overlapping contributions in the keV energy range from the dominant $^{62}$Ni content in the sample were difficult to disentangle from the capture-resonances of interest in $^{63}$Ni (see Fig. 2 in [64]). In short, the small cross section ($\sim$67 mb at 30 keV), the reduced number of $^{63}$Ni atoms (10$^{21}$ atoms, 112 mg) and the need to use a PEEK sample encapsulation contributed to a sample-scattered neutron-induced background which, in a similar way as in the measurement of $^{93}$Zr($n,\gamma$), limited the R-matrix analysis of the resolved resonances from thermal neutron energy up to E$_n\sim$10 keV. The resulting MACS at $kT=30$ keV was of 66.7(13.5) mb [64]. The $^{63}$Ni($n,\gamma$) measurement was complemented with the measurement of the stable $^{62}$Ni [65] for a more reliable evaluation of the impact of these cross sections in the abundance ratio of $^{63}$Cu:$^{65}$Cu in massive stars (M>8M$_\odot$), which is now predicted to be 40% smaller than what was expected before on the basis of previous cross sections. This result allowed to reduce one of the main uncertainties for the abundances of $^{63}$Cu, $^{64}$Ni and $^{64}$Zn in *s*-process rich ejecta of core collapse supernovae (CCSNe).

Recently, with the aim of having a fully consistent picture of the Ni-Cu-Zn abundances, a new measurement on $^{64}$Ni($n,\gamma$) [66] has been carried out at n_TOF and another one is planned on $^{56}$Fe($n,\gamma$) [67]. The measurement of $^{56}$Fe($n,\gamma$) was attempted during the Phase I of n_TOF [68] and, unfortunately, the in-beam $\gamma$-ray background (discussed in Sect. 2) severely constrained the quality of the data. Thanks to the new spallation target design [24] and the improved experimental conditions at EAR1 this cross section will be measured again in the coming years [67]. In the case of $^{64}$Ni($n,\gamma$), the main limitation for a previous measurement was due to the very low natural abundance (0.92%) and the limited sample quantity available, which hindered a measurement in the past at EAR1. Thanks to the high flux and improved RF available now at the EAR2 station (20 m flight path) the $^{64}$Ni($n,\gamma$) cross section has been successfully measured in 2024.

### $^{171}$Tm($n,\gamma$): solving rare-earth element anomalies in meteorites

The difficulty of the $^{63}$Ni($n,\gamma$) measurement was paralleled afterwards by the neutron-capture measurement of $^{171}$Tm (t$_{1/2}$=1.92 year) [69] (see Fig. 2). This experiment was performed at EAR1 utilizing an enlarged setup of four C$_6$D$_6$ detectors for higher detection efficiency. In spite of this, the covered neutron-energy range was limited further down to only $\sim$ 1 keV owing to the much smaller number of atoms in the sample (2$\times$10$^{18}$ atoms). The more limited energy range (1 keV for $^{171}$Tm versus 10 keV for $^{63}$Ni) reflects that the $^{171}$Tm($n,\gamma$) experiment was indeed even more difficult than the previous one on $^{63}$Ni discussed above. In this case, however, the drawback of the small sample quantity was counterbalanced to some extent by the rather large cross section ($\sim$400 mb at 30 keV), the high sample enrichment (98%) achieved at PSI via chemical purification and separation, and the use of four C$_6$D$_6$ detectors, instead of the two units commonly used before. The neutron-capture cross section could be determined in the range from thermal neutron-energy up to $\sim$1 keV, yielding a MACS of 570(220) mb at $kT=30$ keV. In addition, this is one of the relatively few cases where the TOF measurement can be conveniently complemented with an activation experiment which, in turn, became crucial for improving the measurement uncertainty down to 10% at a thermal energy of $kT=30$ keV. The activation experiment was carried out at the SARAF facility [70]. The synergy in this unique combination contributed for building later the new local neutron-activation station (NEAR) at CERN n_TOF [71], which is discussed in Sect. 5.

The $^{171}$Tm result helped to understand striking isotopic anomalies in rare-earth elements (REE) found in bulk SiC grains of the Murchison meteorite, particularly in three samples where the Yb-isotopic composition was analyzed [72]. The $^{171}$Yb/$^{172}$Yb- and $^{173}$Yb/$^{172}$Yb-isotopic ratios could be well reproduced after the new cross sections measured at n_TOF and detailed modeling of the so-called third dredge-ups (TDUs) included in updated stellar models of low-mass stars [73]. SiC grains are thought to be synthesized after TDUs, during which by-products of nuclear burning occurring in stellar interiors, including carbon and *s*-process isotopes, are transported to the surface of the star. Another important consequence of understanding the composition of SiC grains is that nearly half of the carbon-rich envelope’s mass is ejected into the interstellar medium (ISM) by the winds of low-mass AGB stars. The isotopic composition of this ejected material is determined by the last thermal pulse and third dredge-up (TDU) event in these stars [53].

### $^{204}$Tl($n,\gamma$): shedding light on the origin of the heaviest *s*-only nucleus $^{204}$Pb

$^{204}$Tl (t$_{1/2} =3.78$ year) was the latest and most challenging *s*-process branching nucleus measured at EAR1 still with conventional C$_6$D$_6$ detectors [63] (see Fig. 2). The sample contained just 2.66$\times$10$^{19}$ atoms of $^{204}$Tl, representing an enrichment of only 4% with respect to the primary sample isotope $^{203}$Tl. One of the main experimental difficulties was due to a $^{60}$Co-contamination in the sample (373 kBq), which required the unconventional use of a 2 mm thick lead shielding in the front surface of each one of the four C$_6$D$_6$ detectors used for the experiment (see Fig. 3). In addition, a rather high analysis threshold in deposited energy, of 600 keV (more than three times the usual value), was necessary to obtain an optimal signal-to-background ratio. The effect of this cut-off threshold could be nevertheless well accounted for thanks to the methodology developed in Ref. [37], which enables an accurate treatment of such experimental effects by means of realistic Monte-Carlo calculations of the capture $\gamma$-ray cascades and detailed modeling of the experimental setup. The neutron-capture cross section of $^{204}$Tl was measured in the neutron-energy range from thermal up to $\sim$4 keV. At $kT=30$ keV a MACS value of 260(90) mb was determined [63].

The synthesis of $^{204}$Pb is shielded from *r*-process contributions by its stable isobar $^{204}$Hg and thus, the split of the *s*-process path at $^{204}$Tl is the responsible for the existence of all the $^{204}$Pb that we find in the Solar System today. *S*-only nuclei are stable isotopes that, like $^{204}$Pb, are entirely produced by the the *s*-process because they are shielded from *r*-process contributions by stable isobars. From $^{70}$Ge to $^{204}$Pb there exist $\sim$30 *s*-only nuclei across the full nuclear chart. *S*-only nuclides are particularly relevant for benchmarking the performance of TP-AGB models at different metallicities, and for assessing the contribution of such stars to the chemical composition of our galaxy [11]. Previous studies [74] indicated the possible existence of unknown fragmentation mechanisms in the early Solar System affecting the abundance of the lead isotopes in Ivuna-type Chondrites (CI), with respect to the primordial Solar-System abundances. Supernovae are another hypothetical scenario that could potentially affect the abundance of $^{204}$Pb by means of *p*-process contributions [75, 76]. Additionally, addressing the primordial *s*-process origin of $^{204}$Pb is relevant for dating the age of meteorites and their components formed in the first 5 Myr of the Solar System [77]. In particular, Pb-Pb cosmochronometry represents the state-of-the-art for determining the age of the Solar System by dating Calcium-Aluminum rich Inclusions (CAIs) in primitive meteorites [78]. Because $^{204}$Pb is the only lead isotope which is exempt of radiogenic contributions from the decay of U- and Th-isotopes its abundance is used for relative normalization of the radiogenic components ($^{206,207}$Pb) in the Pb-Pb clock (see Eqs. (1–3) in [79]). In this way, $^{204}$Pb becomes key for the unsurpassed $\sim$0.1-0.2 Myr precision [80] of this cosmochronometer. New AGB nucleosynthesis calculations based on the cross section measured at n_TOF delivered $^{204}$Pb abundances fully consistent with the latest solar-system abundance compilation by Lodders [81]. Within the quoted uncertainties this result ruled out the necessity of invoking fractionation mechanisms or any significant *p*-process contribution to the origin of $^{204}$Pb. Reducing further the uncertainty on the *s*-process contribution to $^{204}$Pb will require an accurate assessment of the thermal dependency of the $\beta$-decay rate of $^{204}$Tl, which could be achieved in the next years in the framework of the PANDORA project [82].

Neutron-capture measurements on even more difficult *s*-process branchings utilizing samples with smaller number of atoms, lower enrichment and/or higher $\gamma$-ray activity, required the use and optimization [25] of the second experimental area EAR2 at only 20 m from the spallation source. EAR2 delivers an instantaneous neutron flux, which is more than two orders-of-magnitude larger than the one available at EAR1 [30], making it a unique tool for suppressing the background contribution arising from the sample radioactivity. In addition, developing two new detection systems [40, 42, 44, 83] was necessary for the measurement of the next two, even more challenging, *s*-process branching nuclides $^{94}$Nb and $^{79}$Se, as described below.

**Fig. 4:**
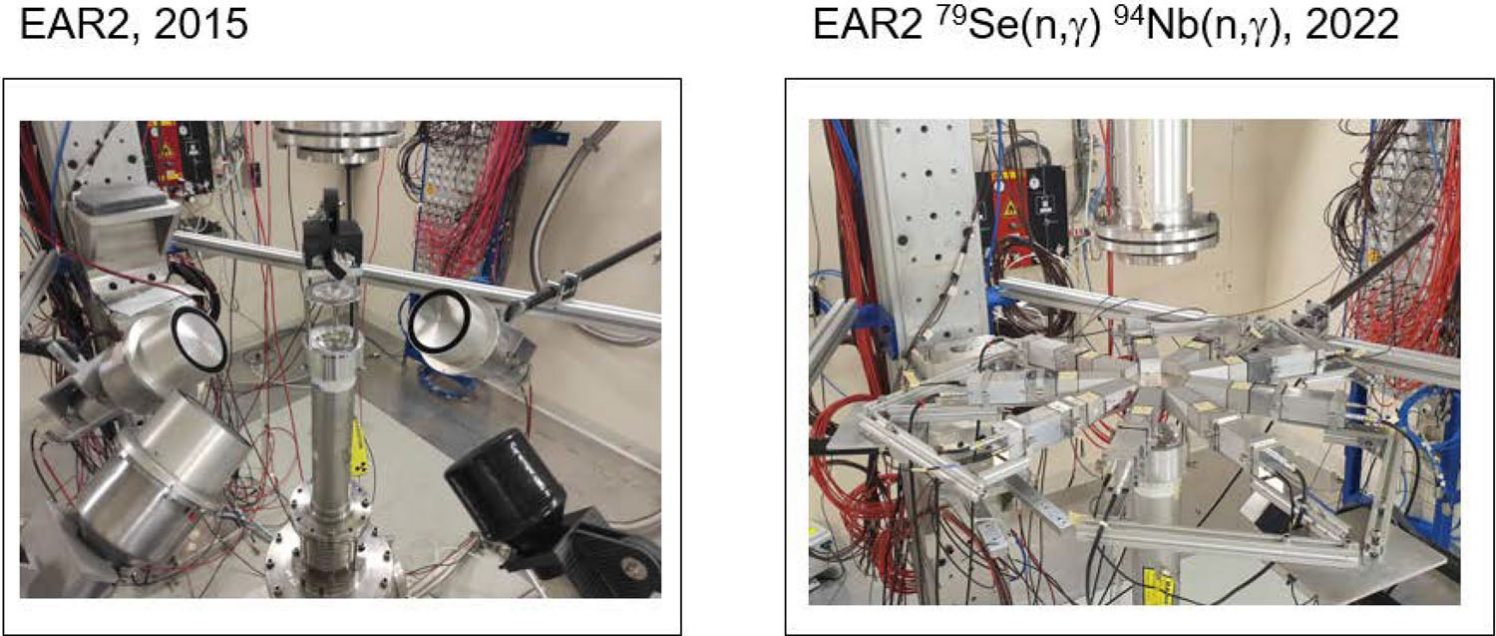
Left: Conventional (large-volume) C$_6$D$_6$ detectors for capture measurements in EAR2. Notice that due to their large volume and the high neutron-flux the detectors had to be placed far from the sample in order to limit the count-rate per detector. Right: Upgraded setup with nine small volume C$_6$D$_6$ sTED detectors in a compact configuration surrounding the capture sample

### $^{94}$Nb($n,\gamma$): disentangling Mo-isotopic anomalies in presolar SiC grains

The sample of $^{94}$Nb (t$_{1/2} = 2\times 10^4$ year) contained 9$\times$10$^{18}$ atoms with only an 1% enrichment with respect to $^{93}$Nb. In addition, the sample itself had a $^{60}$Co contamination of 10 MBq. These challenging features, exceeded the experimental capabilities of EAR1 (see Fig. 2) and hindered the use of conventional C$_6$D$_6$ detectors (see Fig. 4-left). The capture yield of the $^{94}$Nb($n,\gamma$) reaction could be successfully measured up to 10 keV of neutron energy implementing in EAR2 a new array of nine small-volume C$_6$D$_6$ (sTED) detectors [44, 45] in a compact configuration around the sample (see Fig. 4-right). The high instantaneous flux of EAR2 and the new set-up of detectors optimized for very high count-rate capability ($>10$ MHz) became crucial for overcoming the limitations imposed by the $^{60}$Co sample activity (see Fig. 6 in Ref. [83]).

The neutron capture-cross section measurements of $^{94}$Nb at n_TOF and other stable Mo-isotopes discussed below, were motivated by a better understanding of AGB-contributions to the composition of mainstream SiC grains. Mainstream grains arise from low-mass C-rich AGB stars, as it is inferred from their isotopic signature [84] and the presence of SiC dust around such stars [85]. The heavy-element isotopic compositions of mainstream grains, like Mo-isotopes, is less affected by initial stellar composition or GCE effects as compared to light-elements (A<56) and, for this reason, these elements are especially well suited for the study of AGB-stellar processess [86]. The Mo-isotopic abundances of mainstream grains were generally well reproduced by state-of-the-art AGB models, with the exception of $^{94}$Mo [53]. There are several reasons that could affect the production of $^{94}$Mo in stars, among them the branching at $^{94}$Nb (see Fig. 1 in [53]) leading to the formation of $^{94}$Mo. This contribution is enhanced during the He-shell flashes of TP-AGB stars by the reduced $^{94}$Nb half-life of only a few days at *T* = 3$\times$10$^8$ K [87]. Uncertainties on the thermal-dependency of the $^{94}$Nb $\beta$-decay rate, and the unknown $^{94}$Nb($n,\gamma$) neutron-capture cross section could be responsible of the observed discrepancy [53]. Low-mass TP-AGB models [53] predict about 4 times less $^{94}$Mo than the relative quantities found in SiC grains (see Fig. 8 in [53]). More recent low-mass AGB models with updated magnetohydrodynamics-based mixing schemes [88, 89] also yield an anomalous difference between predicted and measured $^{94}$Mo abundance ratios in mainstream-SiC grains (see Fig. 8 in [90]). Among the different isotopes involved, obviously neutron-capture on the unstable $^{94}$Nb is the most difficult one to measure owing to the reasons highlighted above. However, uncertainties in the cross sections of the stable Mo-isotopes should not be ruled out and, for that reason, additional measurements on the stable $^{94-96}$Mo isotopes have been recently carried out at CERN n_TOF EAR1 [91, 92]. Both the data-analysis of $^{94}$Nb($n,\gamma$) and $^{94-96}$Mo($n,\gamma$) are presently in progress and, once finalized, the uncertainties from the neutron-capture input data should be removed from this problem. It remains to be investigated if a different thermal dependency of the $^{94}$Nb $\beta$-decay rate in the stellar plasma conditions could have a significant impact. Fortunately, the measurement of this decay-rate at high (stellar) temperature is precisely one of the first priorities of PANDORA [82].

### $^{79}$Se($n,\gamma$): temperature in Massive and AGB stars

Following the FOM defined in Eq. (1), the most challenging *s*-process branching measured so far at CERN n_TOF via the time-of-flight technique is $^{79}$Se (see Fig. 2). $^{79}$Se has a terrestrial half-life t$_{1/2}$ = 3.27(8)$\times$10$^5$ years [93] and the chemical element selenium itself has a melting point of only 494 K. Because of such low melting point an eutectic lead-selenide (PbSe) alloy [94], highly enriched in $^{208}$Pb and $^{78}$Se, was prepared at PSI with a total mass of 3.9 g [95] for subsequent activation at the high-flux reactor of ILL-Grenoble. The eutectic alloy was necessary to comply with safety regulations at ILL. The resulting activated sample contained only 2.7 mg of $^{79}$Se, thus corresponding to an enrichment factor as low as 7$\times$10$^{-4}$. In addition, the activity due to the $\gamma$-ray emitting decays of $^{75}$Se and $^{60}$Co from ineluctable impurities activated in the sample were of 5 MBq and 1.4 MBq, respectively. Finally, the high Pb content in the PbSe sample significantly enhanced the background due to elastic neutron-scattering events in the sample itself. The scattered neutrons were captured in the surrounding materials, emitting $\gamma$-rays that further worsened the experimental conditions. To address this challenge, a novel detection system, i-TED [40], was developed. i-TED combines the TED principle with advanced $\gamma$-ray imaging techniques [41, 42, 96, 97]. The imaging capability helped suppress localized $\gamma$-ray backgrounds arising from contaminant neutron-capture events in the surrounding materials, thereby enhancing detection sensitivity for the $^{79}$Se($n,\gamma$) channel of interest [43, 98].

The complex data-analysis related to the use of the i-TED system [40, 42] in EAR1 (see Fig. 3) and the sTED detector array [44, 83] in EAR2 (see Fig. 4) is expected to be completed soon. We clearly observe a number of resonances below $\sim$1 keV related to neutron-capture on $^{79}$Se which means that, once the data analysis is completed, it will be possible to provide the very first neutron-capture nuclear-input required for a proper interpretation of this important *s*-process branching point.

**Fig. 5:**
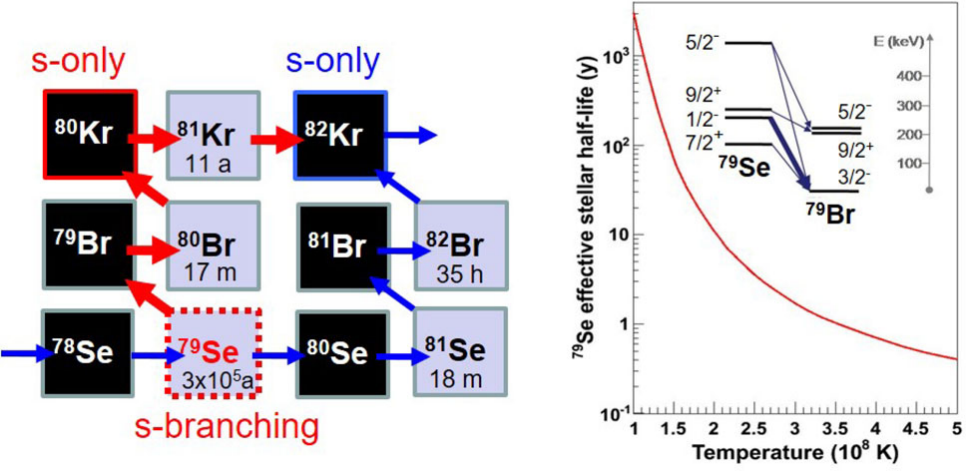
Left: Part of the nuclear chart showing the *s*-process path at the branching in $^{79}$Se. The $\beta$-decay towards *s*-only $^{80}$Kr is strongly enhanced at higher stellar temperatures (red arrow) due to the population of the $1/2^-$-isomeric state at low energy. Right: Effective half-life of $^{79}$Se as a function of stellar temperature. The inset indicates levels involved

The branching at $^{79}$Se represents indeed one of the best cases to constrain the thermal conditions of the *s*-process [7, 99]. Under stellar conditions, the half-life of $^{79}$Se decreases to less than a year due to the population of the isomeric state at only 95.7 keV [87, 100] (see Fig. 5). Remarkably, this subtle nuclear-structure effect impacts the final abundances for the *s*-only $^{80,82}$Kr isotopes, which are strongly dependent upon the thermal conditions inside the star and the neutron-capture rate at $^{79}$Se. Moreover, this neutron-capture cross section measurement could provide valuable information about *s*-process nucleosynthesis both in massive stars and in AGB stars because the *s*-process path induced by $^{79}$Se is located in the transition region between the weak (in massive stars) and the main (in low-mass AGBs) *s*-process contributions [99]. The stable end-products of the Se branching, the (*s*-only) Kr isotopes, have been well characterized in presolar graphite grains of the CM2 Murchison chondrite [101]. A striking property of these graphite grains is that their isotopic features depend on their density and, in fact, low-density grains are inferred to predominantly arise from CCSN explosions of massive stars [102, 103], while high density (>2.1 g/cm$^3$) graphite grains are thought to mainly originate from C-rich AGB stars [101]. Therefore, once the experimental result from the neutron-capture cross section measurement carried out at n_TOF becomes available in the near future, a complete assessment of the thermal conditions both in AGB and massive stars should become possible by revisiting the readily available Kr-isotopic abundances from presolar low- and high-density graphite grains extracted from the Murchison meteorite.

## Recent measurements on stable isotopes

Although neutron-capture cross-section measurements exist for all stable isotopes of *s*-process interest, the related uncertainties are in most cases far from the ±5% uncertainty level required for *s*-process model calculation utilizing low-mass AGB models [73, 104, 105] and massive-star models [106, 107]. Significant progress has been made in recent years to study the challenging unstable s-process nuclei. However, many key aspects of stellar nucleosynthesis in AGB and massive stars remain uncertain and still require extensive research with stable isotopes. Future efforts with stable nuclei should prioritize new neutron-capture measurements with enhanced accuracy and covering the full energy range of astrophysical interest. In the following some recent examples are discussed in more detail.

### *S*-process bottlenecks: $^{140}$Ce($n,\gamma$) and $^{209}$Bi($n,\gamma$)

The neutron shell-closure effects along the *s*-process path are reflected in small capture cross sections, which lead to characteristic abundance peaks in the mass-regions of Sr, Y, Zr (N = 50), Ba, La, Ce, Nd, Sm (N = 82) and Pb, Bi (N = 126). Because *s*-process bottleneck elements, in general, show-up prominently in spectroscopic observations of stellar atmospheres, from the observational point of view they can be accurately quantified in different types of stars, thus becoming good candidates for testing the details of the stellar models. However, cross section uncertainties for a large number of neutron-magic nuclei are still very sizable (see Fig. 6 in [8] and Fig.1 in [108]). An uncertainty below $\sim$2% [7, 109] would be more convenient for a proper interpretation of the observed abundances and for exploring different aspects of low-mass AGB stars, such as the effect of metallicity [110] or the amount of $^{13}$C in the pocket [13, 49, 111].

In practice, experiments with neutron-magic nuclei often present significant challenges due to their extremely low radiative capture cross sections. In some cases, these cross sections barely exceed a few millibarns at $kT = 30$ keV [27, 112, 113]. Consequently, the radiative capture cross section can be up to three to four orders of magnitude smaller than the competing elastic scattering one within the stellar energy range of interest. In these scenarios, the high number of scattered neutrons may interact (promptly or after partial thermalisation) with materials in the surroundings of the experimental setup and detectors themselves. Such contaminant interactions emit radiation that contaminates the measurement and increases background levels. This phenomenon, referred to as neutron sensitivity, has driven advancements in detection systems. C$_6$D$_6$ detectors were introduced as a more suitable alternative to the C$_6$F$_6$ detectors commonly used in earlier studies [114]. At n_TOF, carbon-fibre based C$_6$D$_6$ scintillation detectors were initially developed [38] and progressively improved after many years of operation [39], thereby aiming at minimizing potential neutron sensitivity backgrounds (see Fig. 3). These developments were of particular importance for achieving a high systematic accuracy in the measurement of neutron-magic isotopes [27]. Another source of background that may significantly affect these measurements is the in-beam $\gamma$-rays background, which becomes enhanced when measuring high-Z samples. In summary, the very-low capture cross sections of bottleneck isotopes make their measurement very sensitive to any background contribution, and especial care needs to be taken in the design of the detection set-up and in the detectors themselves. Two recent *s*-process bottleneck measurements will be discussed here: $^{140}$Ce [113] and $^{209}$Bi [115].

### $^{140}$Ce: the second *s*-process peak

For the measurement of the $^{140}$Ce($n,\gamma$) cross section [113] a highly enriched $^{140}$Ce sample was produced at PSI. The sample mass was of 12.318 g (Ce-oxide) and the amount of $^{142}$Ce was of only 0.6%. This high-quality sample enabled a high-resolution measurement at EAR1 covering a large neutron-energy range up to 65 keV. The conventional setup of four C$_6$D$_6$ detectors optimized for low neutron sensitivity was utilized. The resulting MACS at $kT=30$ keV was of 13.15(39) mb. For nucleosynthesis in AGB stars the MACS at $kT=8$ keV is more relevant, which was found to be of 28.18(24) mb, almost 40% higher than expected [116]. These results, in combination with low-mass AGB stellar models [73, 117] yielded a reduction of 20% in the *s*-process abundance of $^{140}$Ce with respect to previous estimations.

The $^{140}$Ce($n,\gamma$) experiment was motivated by a discrepancy of 30% found between the Ce abundance predicted with AGB models and the quantity determined from spectroscopic observations of stars in the M22 globular cluster, for which the *s*-process pollution could be well isolated [118]. Interestingly, a good agreement was found between abundance predictions and observations for the neighbouring bottleneck elements: Ba, La, Nd and Sm (see Fig. 11 in [118]). The new cross section leads to an even smaller predicted Ce-abundance (see Fig. 1 in [113]), which does not solve this discrepancy and makes this problem even more intriguing. In addition, other independent activation experiments [119] provide a MACS value significantly lower (MACS = 9.7(5) mb at $kT=$30 keV) than the TOF result. This situation reflects the inherent difficulty in the measurement of bottleneck isotopes, which are characterized by their very low cross sections. As a consequence, a new activation experiment has been carried out at the new NEAR facility (see Sect. 5) of n_TOF utilizing the same high-quality sample that was employed for the TOF experiment. For completeness, it is worth mentioning that some impact on the observed abundance may come from possible *i*-process contributions [120, 121], that could be enhanced by a rather small $^{140}$Ba($n,\gamma$) cross section. Obviously, this hypothesis is very uncertain and in any case it cannot resolve the discrepancies in the MACS determined from different experiments. Both theoretical, experimental and (probably) observational efforts seem to be required at this stage in order to shed more light on this interesting case.

### $^{209}$Bi: the third *s*-process peak

Bismuth is the heaviest element produced by the *s*-process in low-mass AGB stars, it is monoisotopic (A = 209), and its abundance in the Solar System is relatively well known [81]. The $^{209}$Bi($n,\gamma$) cross section was measured at CERN n_TOF EAR1 in 2001 [27] with a set-up similar to the one shown in Fig. 3-left. The MACS for $^{209}$Bi($n,\gamma$) was found to be of only 2.89(50) mb at $kT=25$ keV, which corresponds to the lowest cross section among all the examples reported in this article, and the second smallest measured at CERN n_TOF only after $^{25}$Mg($n,\gamma$) [112]. Together with the measurement of the neighboring lead isotopes $^{204,206,207}$Pb [122–124], relevant information could be obtained for the study of the termination region of the *s*-process path (see previous references and Sec.5 in Ref. [125]). In particular, rather accurate abundance constraints could be derived for the *r*-process contribution to $^{209}$Bi (see Fig. 5 in [125]) via the so-called *r*-process residuals ($N_r = N_\odot - N_s$), given that the corresponding *s*-process abundance was well determined from the measured cross section and TP-AGB stellar models (see [27] and references therein) and the fact that the solar system abundance of Bi ($N^{Bi}_\odot$) is relatively well known ($\sim$7% relative uncertainty) from the isotopic analysis of CI-chondrites [81]. In turn, the *r*-process residuals analysis helped to qualify or reject some of the former nuclear-mass models utilized in *r*-process model calculations [125, 126].

**Fig. 6:**
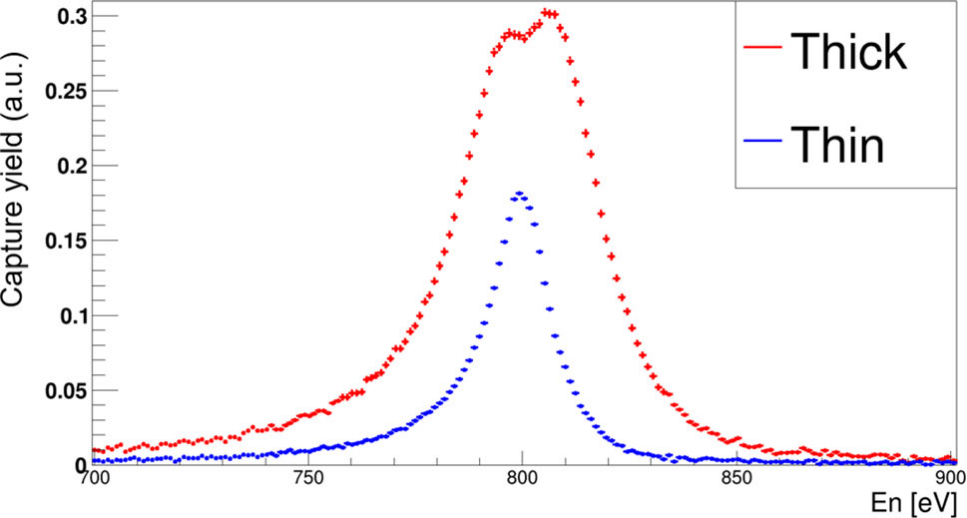
Comparison of the capture yields measured at n_TOF EAR2 [115] for the first capture-resonance in $^{209}$Bi+n with a thick and a thin sample. The double-peak structure arises from enhanced multiple neutron-scattering effects inside the thick sample

However, despite efforts in reducing the intrinsic neutron-sensitivity of the detection apparatus, the first measurement of $^{209}$Bi at n_TOF encountered many difficulties due to the aforediscussed in-beam $\gamma$-ray background, which was significantly enhanced by the high-Z (83) of the sample. This background was indeed largest in the $\sim$10 keV neutron-energy range, which hindered the observation of some resonances and reduced significantly the statistical accuracy in many of them. With the new improved experimental conditions discussed in Sect. 2 a new measurement of $^{209}$Bi($n,\gamma$) was successfully carried out in 2024 at EAR2 [115, 127]. Preliminary results indicate that with the new measurement one could obtain an improvement in both the covered energy range and the number (and statistical accuracy) of resonances observed. In addition, the high-flux at EAR2 enabled the measurement of two independent bismuth samples, with two different thicknesses of 6 mm and 1 mm, which permitted a better assessment of multiple-scattering and neutron-sensitivity effects. These two experimental effects are of particular importance in the case of broad s-wave resonances (see Fig. 6).

### $^{28-30}$Si($n,\gamma$): disentangling Si-Ti-Mg isotopic anomalies in mainstream SiC grains

Even for the most abundant and best known mainstream SiC presolar grains, already discussed here in the context of the $^{94}$Nb-branching in Sect. 3, the mass and metallicity properties of their progenitor AGB stars are still quite ambiguous [128, 129]. Modern galactic chemo-dynamical models indicate that C-rich AGB stars with masses $M \sim 2 M_\odot$ and metallicities $Z\sim Z_\odot$ seem to be the main progenitors of these SiC grains in our Solar System [128], whereas previous studies rather point to AGB stars with masses up to 4 $M_\odot$ and $Z\sim 2 Z_\odot$ [129]. In turn, the mass- and metallicity-distributions of the AGB-star population is of fundamental relevance for *s*-process nucleosynthesis and for understanding the chemical evolution of our galaxy. As discussed before in the context of the $^{94}$Nb branching in Sect. 3, heavy element isotopic compositions of mainstream grains, like Zr or Mo, can help to avoid effects related to the ISM composition or the imprints of GCE. In practice, improving current uncertainties in the neutron-capture cross sections of the light Si-isotopes, commonly used for the isotopic-classification and interpretation of SiC grains [86], may also contribute to disentangle possible imprints of the ISM composition in the formation of these grains from the intrinsic nucleosynthesis contribution of the C-rich AGB progenitor.

**Fig. 7:**
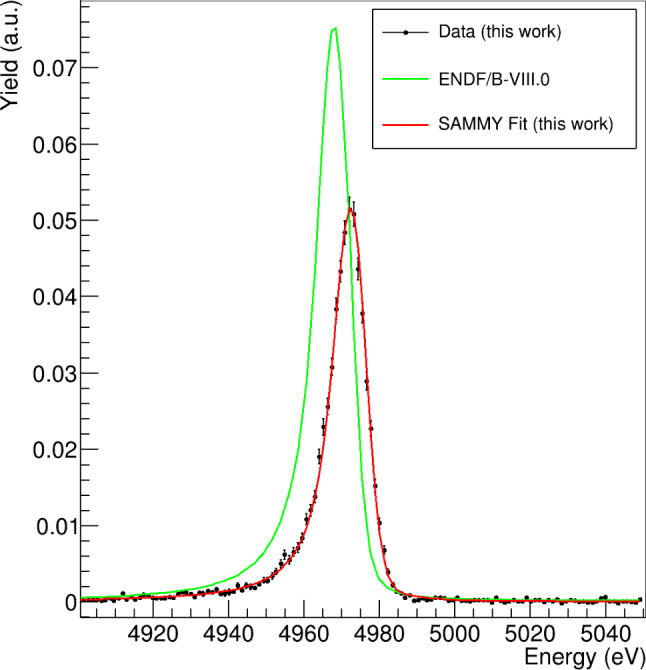
R-matrix fit and its expected shape based on the ENDF/B-VIII.0 parameters of the first resonance in $^{30}$Si+n measured in EAR1

Experimentally, neutron-capture measurements on silicon isotopes are challenging owing to their very small neutron capture cross section. A further difficulty is the significant direct capture contribution expected in these light nuclei, which can be estimated theoretically or inferred from the thermal neutron-capture value if it is precisely known. Latest neutron-capture measurements on the Si-isotopes were rather inconclusive regarding the interpretation of the isotopic-composition of mainstream SiC grains, with important shifts with respect to expectations found for $^{29,30}$Si [130]. In an effort to improve this situation a new series of measurements on $^{28,29,30}$Si$(n,\gamma )$ were carried out at n_TOF utilizing highly enriched samples, state-of-the-art C$_6$D$_6$ detectors and both EAR1 and EAR2 experimental areas [131]. The two independent measurements in EAR1 and EAR2 with complementary setups (four C-fiber C$_6$D$_6$ and nine sTED-detector array) allow one to keep under control systematic effects related to multiple-scattering and other experimental effects. The R-matrix analysis of the capture yield for all the measured Si-isotopes is presently in progress. In the case of $^{30}$Si($n,\gamma$) a number of resonances have been observed up to several hundreds keV and the MACS at 30 keV seems to be dominated by the first strong resonances in the 5–15 keV energy range. Fig. 7 displays an example of the excellent statistical quality of the data, showing a capture yield that is remarkably smaller than the values predicted by evaluations.

## Present limits and new horizons

Some of the main persistent issues and limitations in the field of neutron-capture experiments for *s*-process studies are discussed in this section. A synopsis is also given on current plans for overcoming such limitations in the coming years at n_TOF, as well as new envisioned ideas for advancing further in a longer-term plan (coming decades).

The very first limitation in most of the challenging cases shown in Fig. 2 starts with the quality of the capture sample itself. In spite of efforts and resources invested in manufacturing the best suitable samples for capture experiments, in most cases their final quality and composition still represent one of the main restrictions. Let us take as an example the most challenging $^{79}$Se($n,\gamma$) case discussed in Sect. 3. Having a chemically pure sample of Se, instead of a lead-selenide eutectic alloy, would represent a remarkable advantage. All backgrounds arising from neutron-scattering in the lead content would be removed. Additionally, the $^{60}$Co contamination in the measured PbSe sample (1.4 MBq) arose from traces of $^{nat}$Co in the highly enriched $^{208}$Pb raw material used for producing the eutectic PbSe alloy [95]. Such contamination could be avoided with a chemically pure sample of Se. It is technically feasible to obtain a radiochemically pure sample of selenium, such as the sample prepared at PSI for the measurement of the $^{79}$Se half-life exploiting reductive deposition, anion exchange chromatography and sublimation [132]. The latter sample contained only Se-isotopes (see Table 6 in Ref. [132]), including a significant amount of $^{79}$Se. However, the cost and resources required to obtain a pure Se-sample with a sufficient number of $^{79}$Se atoms (>10$^{18}$) for a TOF experiment would be prohibitive. A further remarkable improvement could be obtained with a radio-isotopically pure sample of “only” $^{79}$Se, which would allow one to reduce backgrounds and capture on sample impurities. Such a sample could probably lead to an experimental determination of the neutron-capture cross section in the full energy range of astrophysical interest ($\sim$1 eV–100 keV). This additional purification step would require the use of a dedicated mass-separator and the final sample amount would strongly depend on the design of such apparatus and the initial quantity and quality of material available. Only a few laboratories worldwide are equipped with the instrumentation and knowledge required to handle and produce such samples [133]. A big coordination effort is being pursued in the context of the SANDA [134] and its follow-up APRENDE project [135], as well as by the International Nuclear Target Development Society [136]. An off-line isotope separator for such purpose has been envisaged at PSI and it would be a unique tool for the production of radio-isotopically pure samples with sufficient amounts, in suitable backings and free from oxides (see also related discussion in Ref. [137]).

Another major limitation of TOF experiments, as evident from the analysis of the seven *s*-process branching examples discussed in Sect. 3 (see Fig. 2), is the limited neutron-energy range they can efficiently cover. The white neutron-flux spectrum and low duty-cycle of n_TOF allows one to fully cover the 1 eV to 100 keV energy range of interest for *s*-process studies. However, that wide energy range could only be analyzed in the very first $^{151}$Sm($n,\gamma$) experiment (see Fig. 1 in Ref. [26]). For all other *s*-process branching cases discussed in Sect. 3, the upper data-analysis limit was between 1 and 10 keV, thus missing an important (if not the most important) part of the stellar spectrum. In many cases the energy range beyond 10 keV corresponds already to the unresolved resonance region (URR) and thus, if the signal-to-background ratio is not adequate, it becomes increasingly difficult and uncertain to reliably extract the cross section. In such cases average resonance parameters were determined in the measured (low-energy) range, and a simulation of random-resonance sequences was computed to determine the MACS at higher *kT* thermal energies (see e.g. [63, 69]).

Improving the experimental background conditions, or enhancing further the sensitivity of the detection apparatus with ideas similar to i-TED [40, 42] or sTED [44] could help to overcome this limitation in future experiments. Presently, there are several efforts in this direction. A new (enlarged) version of the sTED array is being developed for experiments at both EAR1 and EAR2, thereby exploring a new technique with intermediate detection efficiencies [138]. Moreover, a new version of the sTED-detector array based on stilbene-d$_{12}$ organic crystals instead of C$_6$D$_6$ cells [44], the so-called Stilbene-d$_{12}$ deTector ARray (STAR) [45], has been designed and is already under construction. The use of smaller detection volumes ($25\times 25\times 50$ mm$^3$) but even with a higher intrinsic detection efficiency than liquid C$_6$D$_6$ is expected to enhance further the signal-to-background ratio in future experiments at EAR2. It is foreseen that STAR will be commissioned in 2026 and operational for first capture experiments in 2028, after the third long shutdown (LS3) of CERN.

**Fig. 8:**
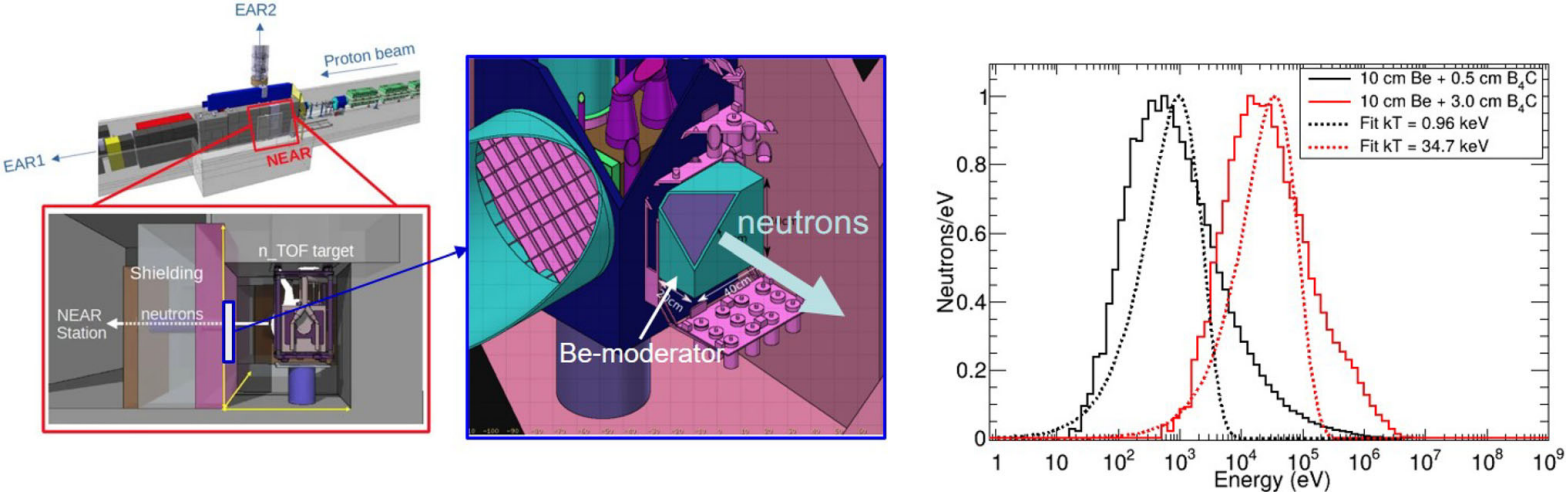
Drawings showing the location and different elements of the new NEAR station (left) and some quasi-Maxwellian neutron distributions obtained via MC-Simulation for different B$_4$C- filter thicknesses, leading to mean kT values of about 1 keV and 35 keV

A complementary and alternative effort, that in some specific cases may help to overcome the aforementioned limitation in neutron-energy range is the new NEAR activation station [71, 139, 140], located at only 5 m from the spallation source (see Fig. 8). With an average flux of $\sim$5$\times$10$^7$ n/cm$^2$/s this new facility will open the possibility to conduct, when feasible, activation experiments with quasi-Maxwellian neutron distributions spanning from $\sim$keV up to $\sim$100 keV. The precise value of *kT* will be determined by the chosen (variable) thickness of a boron carbide (B$_4$C) filter embedding the sample under study [139]. A commissioning of the NEAR station and a flux characterization has been already carried out [139] and its main features and ancillary equipment are described in detail in Ref. [71]. A Be moderator directly attached to one side of the spallation target will be installed during 2025, which will allow to improve the quality of the quasi-Maxwellian neutron spectrum significantly (see insets in Fig. 8). The characterization and validation of the activation facility in 2026 will allow one to determine the main performance features for activation experiments.

The potential of NEAR for new activation measurements on unstable nuclei will be boosted by the availability of the nearby ISOLDE facility and the large yields that can be produced therein [141, 142]. Radioisotopically pure samples with sufficient number of atoms ($\sim$10$^{15-16}$) will be produced at ISOLDE for the envisaged activation experiments at n_TOF NEAR. At present, this approach is already being tested with the production of a $^{135}$Cs sample at ISOLDE utilizing General Purpose Separator (GPS) [143]. Once the sample becomes available, the activation measurement at NEAR is foreseen for 2026 [144]. For the measurement of isotopes leading to short-lived activated products the CYCLING (CYCLIc activation station for (N,G) experiments) is being prepared and background characterization measurements at NEAR have been already carried out utilizing active (NaI and LaBr$_3$) scintillation detectors [145]. With NEAR and NEAR-CYCLING, in combination with the enhanced sample-production capabilities of ISOLDE, ILL and PSI, we expect to expand the number of ($n,\gamma$) cases of astrophysical interest in the coming years, as well as to provide complementary information in the 10–100 keV energy range, for those TOF experiments in EAR1 or EAR2 that are technically restricted to maximum neutron-energies of $\sim$10 keV. Several *s*-process cases to be tackled in the future at NEAR have been discussed already in Refs. [25, 108], as well as some direct measurements for *i*-process studies.

In the longer term, the n_TOF collaboration is planning to expand further on the large potential of the activation technique and on its complementarity for many TOF experiments carrie out in EAR1 and EAR2. In this respect, an expression of interest for a new high-flux activation station n_ACT has been prepared [146]. The latter would be built at the SPS Beam Dump Facility (BDF) [147], where ultra-high neutron fluxes will be produced. n_ACT would allow to strategically profit such high neutron fluence. Preliminary designs for n_ACT [146] predict about three orders of magnitude higher fluxes than what is presently achievable at NEAR. Again, in combination with radio-isotopically pure samples from ISOLDE, this new facility may open-up the possibility to access for the first time direct (activation) measurements on samples available in minor quantities, including some neutron-rich unstable nuclei in the *i*-process path.

Finally, regarding possible long-term advancements, it is worth discussing here the concept of direct neutron-capture measurements in inverse kinematics with a stationary neutron target, which was proposed in Refs. [148, 149]. If feasible, this novel methodology will allow one to overcome experimental difficulties that have been discussed above, mainly those connected with the production of the capture sample, the relatively large number of atoms required, the half-life of the isotope of interest and the radioactivity of the sample itself. Other additional aspects related to detector-sensitive backgrounds could be significantly improved as well. This new technique involves the combination of three main elements, namely, a radioactive ion-beam facility (preferably ISOL-type for low-energy secondary beams in the stellar energy range of interest 50-500 keV), a suitable low-energy storage ion-ring, and a heavily moderated spallation neutron source to drive the free neutron-gas target. A neutron target demonstrator is already being developed at Los Alamos Neutron Science Center (LANSCE) [150] and there are plans to demonstrate its performance in the coming years with a proof-of-concept experiment utilizing single-pass stable-beam experiments [150]. At TRIUMF-Vancouver, there are also plans to exploit the high-intensity ISOL yields of the ARIEL facility in combination with a low-energy storage ring (TRISR) and an alternative approach based on compact neutron generators for the static neutron target is under study [8]. One important aspect to take into consideration with this novel methodology is the identification of the the neutron-capture product, with only one mass unit difference (A+1) with respect to the main revolving beam (A). The neutron-capture product has the same momentum of the primary beam, but its speed is reduced by a factor A/(A+1). Thus, a possible solution to this challenge has been studied by means of ion-beam optical calculations by the TRIUMF group [151], and it is based on the use of a velocity (Wien) filter in combination with a recoil separator. With the new inverse kinematics method, direct measurements of neutron-capture cross sections at different neutron-energy values should be also feasible by changing the ion-beam energy.

Interestingly, most of the three basic elements required for the new methodology are readily available at CERN, or at least a large expertise exists with them. On the one hand, ISOLDE would be an ideally well suited installation for the production of low-energy neutron-rich nuclei with high yields for this application. On the other hand, the 25 years of n_TOF experiment have led to a great expertise in the design and operation of spallation-targets [152] (see Fig. 1), including also the new plans at the SPS-BDF facility [146]. A low-energy storage ring, the Test Storage Ring (TSR) was built [153] and operated at MPI-Heidelberg, with later (unsuccessful) plans to install it at HIE-ISOLDE [154]. More recently, in the framework of the EPIC ISOLDE upgrade, there is a renewed interest for a new Isolde Storage Ring (ISR) [155], which could become a reality at CERN in the coming decade.

In summary, after 25 years of groundbreaking developments and remarkable discoveries at CERN n_TOF, our understanding of stellar environments where the *s*-process drives nucleosynthesis has significantly advanced. Looking ahead, innovative approaches such as the inverse-kinematics concept have the potential to redefine the field, paving the way for a new era of research. This methodology could enable the measurement of the few remaining *s*-process branching nuclei, expand studies to most isotopes along the *i*-process path, and even explore regions near the *r*-process path, further bridging the gaps in our knowledge of nucleosynthesis.

## Data Availability

This manuscript has no associated data. [Author’s comment: The datasets generated during and/or analysed during the current study are available from the corresponding author on reasonable request.]

## Code Availability Statement

This manuscript has no associated code/software. [Author’s comment: Code/Software sharing not applicable to this article as no code/software was generated or analysed during the current study.]
